# Text Message Intervention to Facilitate Secure Storage and Disposal of Prescription Opioids to Prevent Diversion and Misuse: Protocol for a Randomized Controlled Trial

**DOI:** 10.2196/60332

**Published:** 2025-04-17

**Authors:** Kathleen L Egan, Melissa J Cox, Donald W Helme, J Todd Jackson, Alice R Richman

**Affiliations:** 1 Department of Implementation Science Division of Public Health Sciences Wake Forest University School of Medicine Winston-Salem, NC United States; 2 Department of Health Behavior Gillings School of Global Public Health University of North Carolina at Chapel Hill Chapel Hill, NC United States; 3 Department of Communication College of Communication and Information University of Kentucky Louisville, KY United States; 4 Pharmacy Services Cape Fear Valley Health System Fayetteville, NC United States; 5 Department of Health Education and Promotion East Carolina University Greenville, NC United States

**Keywords:** prescription opioid, storage, disposal, text message intervention, randomized controlled trial, mobile phone

## Abstract

**Background:**

Nonmedical use of prescription opioids remains a critical public health issue; 8.5 million people in the United States misused opioids in 2022. Most people obtain prescription opioids for misuse from family or friends. Thus, facilitating secure storage and disposal of opioid medications during and after treatment is needed to prevent medication diversion and subsequent misuse.

**Objective:**

The primary objective of this study is to test the feasibility and efficacy of a novel intervention that uses a persuasive, informational SMS text message reminder system to enhance the impact of secure storage and disposal of unused opioid medications. We hypothesize that the SMS text message intervention will increase secure storage during treatment and disposal of prescription opioids after treatment.

**Methods:**

We will use a 2-arm randomized controlled trial to test the intervention for feasibility and efficacy. Participants (aged 18+ years who have received an opioid prescription in the past 2 weeks) will be randomly assigned to either receive the SMS text message intervention or standard-of-care educational materials. Participants in the intervention will receive 4 SMS text messages related to secure storage and 3 messages related to disposal. All participants will complete baseline, midpoint (day 25), and postintervention (day 45) evaluation surveys. We will test whether receipt of the intervention is associated with two primary outcomes, which are (1) secure storage of prescription opioid medication (locked vs unlocked) and (2) disposal of unused prescription opioid medication (disposed vs not disposed). We will use multiple logistic regression to test the main hypotheses that the intervention will be positively associated with secure storage (locked vs unlocked) and disposal (yes vs no) behaviors, which will allow us to control for demographic variables known to influence the outcomes. This protocol represents the entire structure of the randomized controlled trial.

**Results:**

Recruitment for the randomized controlled trial was launched in April 2024, and data collection was completed in December 2024. The final sample size is 484. Data analyses for the main hypothesis will be completed by May 2025, and the main hypothesis manuscript will be submitted for publication by May 2025.

**Conclusions:**

Results from this study will indicate whether a text message reminder system can increase secure storage and disposal behaviors for individuals who receive opioid medication. This type of intervention has the potential to be integrated into currently used health care delivery systems, such as prescription pickup reminders at pharmacies. Thus, the intervention is scalable across systems of care, thus expanding the reach of secure storage and disposal programs to prevent prescription opioid misuse.

**Trial Registration:**

ClinicalTrials.gov NCT05503186; https://clinicaltrials.gov/study/NCT05503186

**International Registered Report Identifier (IRRID):**

DERR1-10.2196/60332

## Introduction

### Statement of the Problem

Despite efforts in the United States to reduce the number of opioid prescriptions through prescribing guidelines [1,2] and drug monitoring programs [3,4], mortality rates due to the nonmedical use of prescription opioids have remained high since their peak in 2010 [5]. National data estimates that 6.1 million people met the criteria for opioid use disorder and 8.5 million (3%) misused prescription opioids in 2022 [6]. The majority of people obtain prescription opioids for misuse from family or friends, with or without their knowledge [6]. Furthermore, many opioid medications go unused; a meta-analysis of postoperative opioid consumption for acute pain by US adults found that 61% of medications [7] remain after treatment. Unused prescriptions can lead to medication diversion, which is the primary source of prescription opioids for misuse [6]. Facilitating secure storage and disposal of opioid medication during and after treatment is needed to prevent medication diversion.

In an early response to the opioid crisis, the Office of the National Drug Control Policy disseminated a plan that included secure storage and disposal of unused opioid medications as key strategies for prevention [8]. This plan specified the need to educate patients on the proper storage and disposal of prescription medications. The Centers for Disease Control and Prevention recommends that medication is stored out of reach of children and pets and opioid medications should be stored in a locked cabinet or drawer [9]. The US Food and Drug Administration (FDA) endorses multiple methods to dispose of unused prescription opioids when they are no longer needed, including medication take-back days, disposal boxes, mail-back programs, and deactivation kits [10,11]. Currently, the FDA also recommends flushing certain types of medications in the toilet or putting them in the trash if other options are not available [12]. The guidance on flushing unused medications is in contradiction to that provided by the US Environmental Protection Agency [13].

Despite the release of the Office of the National Drug Control Policy plan over a decade ago and guidance on secure storage and disposal from federal agencies [9,10,12,13], evidence suggests uptake of the simple behavior of secure storage and disposal is limited. For example, in a study of a nationally representative panel of 1032 adults who had been prescribed an opioid medication, only 8.6% (n=89) stored their medication in a locked location [14]. Similarly, in a study of 113 patients prescribed opioids for cancer pain, only 15% (n=17) locked their opioids while 36% (n=41) stored opioids in plain sight; however, 73% (n=82) indicated a willingness to store their medications in a locked location [15]. Across multiple studies of individuals prescribed opioids for chronic and acute pain, less than a third reported disposal of unused opioid medications [16-19]. Hence, there is significant room for improvement in opioid medication storage and disposal practices.

There is emerging evidence that a more targeted intervention at medical facilities may enhance the secure storage and disposal of unused opioid medications. Several studies have examined the provision of an educational pamphlet to patients who receive an opioid prescription for acute pain [20-23]. In general, the educational pamphlets contained brief information about nonmedical prescription opioid use and instructions for secure storage and disposal. All studies found statistically significant intervention effects on self-reported storage and disposal of unused prescription opioids [20-23]. That is, a targeted intervention to facilitate secure storage and disposal by patients receiving an opioid prescription improved the uptake of these preventive behaviors. However, the rates of utilization of these practices remained low. Across these studies, secure storage did not improve and 48% (45/86) to 78% (37/170) of patients who received an educational pamphlet still did not dispose of their unused opioid medications, which indicates that additional intervention is warranted.

The high prevalence of opioid prescriptions that are not fully used demands an intervention that can scale widely. The widespread adoption and instantaneous nature of mobile phones make them a promising vehicle for economical and systems-based interventions. Almost all (1442/1502, 96%) of US adults have a mobile phone with SMS capabilities [24], indicating that text interventions have the potential to serve as a universal intervention delivery method. Digital health systems have already been implemented in pharmacies. Many pharmacies have established systems that alert patients via text message when medications are ready to be picked up. Patients report that they prefer to receive health information from medical practices via SMS text messages over other forms of communication [25]. Half of pharmacy patrons already use existing pharmacy-based text reminders and smartphone apps [25]. Thus, delivering reminders about the secure storage and disposal of prescription opioids via SMS text messages represents a scalable intervention across systems of care.

### Theoretical Frameworks

**Figure 1:**
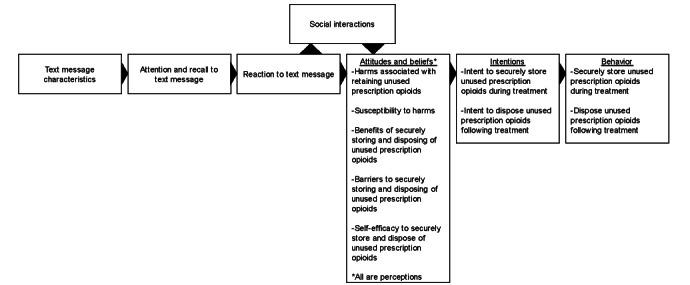
Conceptual framework.

The study is informed by 2 theoretical frameworks (Figure 1). First, the Message Impact Framework [26] suggests that message characteristics affect the extent to which the message will be noticed and later recalled. An individual’s reaction to the message impacts their knowledge, attitudes, and risk beliefs, which in turn impact intentions and actual behavior. Furthermore, exposure to messages can elicit interpersonal communication and social interactions which further spread and influence individuals’ attitudes, beliefs, and reactions to the messages. Second, the Health Belief Model (HBM) [27] posits that messages will generate behavior change if they target perceived barriers, benefits, threats, and self-efficacy specific to the behavior. Specifically, attitudes and beliefs pertaining to perceived seriousness and susceptibility to harms result in the formation of a perceived threat. Along with perceived threats, beliefs about the benefits of and barriers to performing a behavior paired with self-efficacy to do so influence whether an individual will perform the behavior. A cue to action, such as an SMS intervention, serves as a trigger or motivator to perform the behavior, such as securely storing and disposing of prescription opioid medications.

### Objective

The overall objective of the study is to test the feasibility and efficacy of a novel, evidence-informed strategy that uses a persuasive, informational SMS text message reminder system to expand the impact of secure storage and disposal programs. Our central hypothesis is that the implementation of an SMS text message intervention will increase the secure storage of opioid analgesics during treatment and disposal following treatment.

## Methods

### Overview

**Figure 2:**
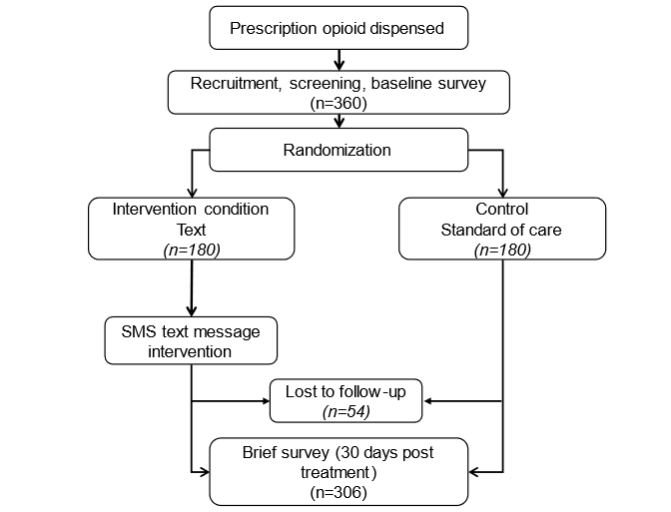
Planned randomized controlled trial study design.

This study uses a 2-arm, single-blinded, randomized controlled trial (RCT) design (Figure 2). Participants are randomly assigned into either the SMS text message intervention condition or a standard-of-care control group. Participants will complete baseline, midpoint (day 25), and postintervention (day 45) evaluation surveys, described further in this study. The primary trial site is Wake Forest University School of Medicine.

### Ethical Considerations

This protocol has been approved by the Wake Forest University School of Medicine Institutional Review Board (IRB; IRB00102139).

The consent form will be self-administered on REDCap (Research Electronic Data Capture; Vanderbilt University). Potential participants will review the consent without the assistance of a study team member and will be directed to the study team if they have any questions. In lieu of a signature, participants will be informed that they should “click ‘I agree to participate’ at the end of the consent form” to provide their authorization to participate in the study. The consent form will use language approved by the Wake Forest University School of Medicine IRB that is designed for readability and includes the general topic of the study, the name of the principal investigator, the principal investigator’s contact information, the IRB approval number, and the phone number of the IRB at Wake Forest University School of Medicine. Participants will be reminded that they are not required to answer questions (other than for eligibility), that they can end participation at any time, and that there is no obligation to participate. Given that participants for the RCT may be patients at a participating institution, they will be reminded during the consent process that participation will not impact their treatment or future access to medications.

To recruit participants, we will maintain a spreadsheet that has identifiable information of individuals who have met the study criteria stored electronically on a secured device and IRB-approved cloud system. The purpose of this spreadsheet is to ensure that potential participants are not invited to participate more than once. After ascertaining consent to participate, unique identifiers will be assigned to participants, and identifiers will be stored separately from the data. To minimize the likelihood of a breach in confidentiality, data will be collected and stored in REDCap, a secure web application for building and managing online surveys, and on a secure and encrypted storage system maintained by Wake Forest University School of Medicine IT security.

All participants in the RCT will be provided with incentives following completion of the baseline survey, midpoint survey, and postintervention surveys, in the form of an electronic US $25 Amazon gift card per survey. Participants will be asked if they are willing to upload a photo of their prescription opioid bottle or box for an additional US $5 Amazon gift card. The maximum amount of money that a participant may receive for participation in this study is US $80. Electronic gift cards will be provided via SMS text message to study participants.

### Participants

#### Eligibility

Individuals are eligible to participate if they are 18 years of age or older, able to read and speak English, own a cell phone with the capability of receiving SMS text messages, within 14 days of being dispensed a prescribed opioid medication, and have an opioid prescription that is for 30 days or less.

#### Sample Size

We plan to enroll 360 participants (n=180 per condition) based on assumptions from an RCT using a text intervention that encouraged parents to vaccinate their adolescent child [28] and an RCT that assessed the delivery of a deactivation product with educational material by medical staff on self-reported disposal of unused opioids [29]. Power calculations based on proportions of vaccine completion for the group exposed to the SMS text message intervention (0.49) and the unexposed group (0.30) [28] indicate a total of 230 participants will be needed to detect a difference in the impact of an SMS text message intervention. Power calculations based on the proportions of disposal for the group who received the deactivation product intervention (0.72) and the unexposed group (0.56) [29] indicate a total of 306 participants will be needed to detect a difference in the disposal of unused opioid medications. We based our sample size on the more conservative estimates [29]. To account for a 15% (12/79) loss to follow-up [21], we plan to recruit an additional 54 participants for a total of 360 participants.

#### Recruitment

We will use a multimethod approach to recruit participants for the RCT. Potential participants will be identified through the Advocate Health electronic health records. Biweekly, we will receive medical record numbers of Advocate Health patients who were recently prescribed an opioid medication. These individuals will be sent an SMS text message via their Advocate Health MyChart inviting them to participate in the RCT. We will also post flyers in local pharmacies with study information. Participants will be directed to a self-administered consent form programmed in REDCap. If the participant consents to participate, they will complete the self-directed web-based eligibility screener.

#### Screening and Randomization

If the individual is eligible to participate in the RCT, they will transition immediately from the eligibility screening questions to the baseline survey questions. Following completion of the baseline survey, a study team member will be notified so they can randomize the new participant to study condition. Randomization will occur within REDCap using simple randomization procedures to randomly assign participants to the intervention or control conditions. Randomization will be stratified by biological sex of the participant. An external module has been integrated into REDCap to confirm that each new participant is unique based on their phone number and email address.

### Intervention

#### Procedures (Intervention Condition)

Participants in the intervention group will receive a series of SMS text messages to securely store prescription opioids during treatment and dispose of unused prescription opioids. Twilio will be used to deliver text messages to participants. Twilio is a third-party web service that integrates with REDCap, allowing users to send survey invitations and alerts or notifications to participants as SMS text messages or voice calls. It acts as a conduit between participants’ mobile devices and the REDCap project. The intervention will last for 45 days and will start immediately following the completion of the baseline survey. All participants randomized to the intervention study condition will receive 7 identical SMS text messages over the course of the intervention. Participants will receive 4 SMS text messages about storing their medications before the midpoint survey which will take place on day 25 of the research study. SMS text messages about the disposal of unused medications will begin after day 31 of the intervention to ensure that all participants would have completed their treatment regimen. Participants will receive 3 SMS text messages about disposing of their unused medications before the postintervention survey, which will take place on day 45 of the research study. Participants will receive 1-2 SMS text messages about storing and disposing of their unused medications per week, reflecting participant feedback from the first phase of this study, which focused on the development of the SMS text message content (Textbox 1) [29]. The order in which all SMS text messages are delivered will be randomized. The recommendations for storing medications have been endorsed by the Centers for Disease Control and Prevention [9], and recommendations for disposal are currently endorsed by the FDA [10].

**Participant-derived SMS text messages.**

**Storage messages**

- It is your prescription, not theirs. Keep your medication hidden and out of reach.
- Locking up your prescription pain pills could save a life. Keep them in a locked location such as a cabinet or box.
- Your favorite hiding spot could save a life. Keep your pain pills where someone would not look for them.
- Your prescription can become someone else’s addiction. Lock up your pain pills.

**Disposal messages**

- Dispose of your unused medications. You may save the life of someone you love. Dispose in a way that works best for you: Return them to the pharmacy, use a home disposal kit, or mix pills with an undesirable substance and put in your trash.
- Discarding your unused pain pills could save a life. Dispose in a way that works best for you: Return them to the pharmacy, use a home disposal kit, or mix pills with an undesirable substance and put in your trash.
- Your prescription can become someone else’s addiction. Safely discard unused or expired medications. Dispose in a way that works best for you: Return them to the pharmacy, use a home disposal kit, or mix pills with an undesirable substance and put in your trash.

#### Control Condition

Individuals who are assigned to the study control condition will receive the standard of care provided to them from their prescribing physician and dispensing pharmacist. Immediately after completing the postintervention survey, participants will receive information on ways to securely store and dispose of unused opioid medication. This information will be provided in the REDCap survey.

### Data Collection and Measures

#### Evaluation

Participants will complete 3 evaluation surveys. Each evaluation will be delivered via a secure link to a web-based REDCap survey. The baseline survey will be completed directly following study enrollment. A midpoint survey will be sent 25 days after study enrollment, and a final postintervention survey will be delivered 45 days after study enrollment. The baseline survey assesses sociodemographic characteristics, information about the prescribed opioid medication, and past medication storage and disposal behaviors. The midpoint and postintervention surveys ask about how the participant has been storing their opioid medication, if they are still using their opioid medication, if they have disposed of their medication, and their intent to dispose of their medication. Participants assigned to the intervention study condition will provide feedback on the SMS text message intervention in the postintervention survey. Those who were assigned to the control study condition will share what they would like to receive in an SMS text message intervention. All surveys will also query perceptions in alignment with the HBM (eg, perceived barriers, and benefits) [27] about securely storing and disposing of their opioid medications.

#### Primary Outcomes

We will use 2 primary outcomes based on data from the postintervention survey. Pertaining to storage of opioid medications, participants will be asked, “Where do you usually store your prescribed pain medication?” with response options of (1) in an unlocked box, closet, cabinet, or drawer; (2) in a locked box, closet, cabinet, or drawer; (3) in a purse, backpack, or other carrier; (4) out in the open; (5) other; or (6) unsure where kept. We will create a binary variable for storage of locked (in a locked box, closet, cabinet, or drawer) versus unlocked (all other responses) to test study hypotheses. Related to disposal behaviors, for participants that indicate they had no leftover medications, we will create a binary variable based on the disposal method of the medication (put in the trash, used deactivation product, used a prepaid mail-back envelope, flushed in the toilet, returned them to the pharmacy, or took them to law enforcement agency) versus not disposed (gave them to friend or family member or something else).

#### Secondary Outcome

For participants who indicate they have medication from their prescription leftover, we will create an intention to dispose variable based on the item “What do you intend to do with your remaining prescription pain medicine?” The binary variable will denote intent to dispose (put them in the trash, use deactivation product, use prepaid mail-back envelope, flush in the toilet, return to pharmacy, or take to law enforcement agency) versus do not intend to dispose (keep them, gave them to friend or family member, or something else).

#### Other Measures

At the baseline and postintervention time points, we will assess measures derived from the HBM including self-efficacy (eg, I know how to properly dispose my prescription medication), barriers (eg, I do not have access to a locked location where I can securely store my prescription pain medication), benefits (eg, disposing my unused prescription pain medications can stop someone else from taking them), perceived severity (eg, I think there are risks to having prescription pain medication in my home), and perceived susceptibility (eg, I worry about having prescription pain medication in my home). At the midpoint and postintervention time points, we will also assess the SMS text messages themselves using items derived from the Message Impact Framework, including whether the participant felt the SMS text messages grabbed their attention, were easy to understand, and made them think about the risks of having prescription pain medicines in their homes.

#### Data Management

Before ascertaining consent to participate, we will maintain a spreadsheet stored electronically on a secured device and IRB-approved cloud system that has the name, phone number, and email address of individuals identified via Advocate Health electronic health records who have met study eligibility criteria. This spreadsheet will include the date we contacted the individual and the way in which we contacted them. The purpose of this spreadsheet is to ensure that an individual is not invited to participate more times than approved by the IRB. This spreadsheet will also have a unique identifier, which will be linked to data pertaining to their prescription number, medication list (ie, opioid prescription only), age, gender, and race or ethnicity. We will retain this information for use in the event that they consent to participate in the study. The spreadsheet will be destroyed once recruitment has closed. To minimize the likelihood of a breach in confidentiality, data will be collected and stored in REDCap, a secure web app for building and managing web-based surveys, and on a secure and encrypted storage system maintained by Wake Forest University School of Medicine IT security. Public access to deidentified data will be made public at the conclusion of the study.

### Data Analysis

#### Design and Data Preparation

Analyses will be conducted to assess group differences on the two primary dichotomous outcomes, which are (1) secure storage of the opioid medication (locked vs unlocked) and (2) disposal of unused medication (disposed vs did not dispose). We will use a modified intention-to-treat (ITT) design. While an ITT design minimizes bias and type 1 error, it is often considered a conservative approach that may increase type 2 error [30]. A modified ITT design allows for the exclusion of some randomized subjects in a justified way to achieve the goal of minimizing both type 1 and 2 errors [30]. For this study, we will retain the ITT approach by including all participants regardless of their compliance with the SMS text message intervention. We will modify the ITT approach by excluding participants who do not have outcome data since we will not have a mechanism to assess their storage or disposal behaviors, which could inflate type 2 error if retained in analyses. We will first test for group equivalence in demographic and household characteristics of the participant, diagnosis and treatment, and information about the prescribed opioid (eg, type, number of pills, and duration of treatment). Group differences are not expected in the context of randomization; however, if one of these variables is associated with both a condition and an outcome variable, we will include it as a covariate in subsequent analyses using that outcome. Data will be compiled and screened for integrity, outliers, missing values, and violations of the assumptions of logistic regression. Missing values will be handled in regression analyses using full maximum likelihood estimation.

#### Statistical Analyses

We will use multiple logistic regression to test the main hypotheses that the intervention will be positively associated with secure storage (locked vs unlocked) and disposal (yes vs no) behaviors, which will allow us to control for demographic variables known to influence the outcomes. We will use a 2-step logistic regression to predict each outcome. In step 1, demographic variables will be entered into the model. We will retain variables significantly associated with the outcome at a significance threshold of α<.05. In step 2, the intervention condition will be entered into the model.

## Results

Recruitment for the RCT was launched in April 2024, and the first participant enrolled in the study in June 2024. The primary completion date, defined as the date on which the last participant in a clinical study completed the postintervention survey, was in December 2024. The final sample size is 484. Data analyses for the main hypothesis will be completed by May 2025, and the main hypothesis manuscript will be submitted for publication by May 2025.

## Discussion

### Contributions to the Literature

The overall objective of the study is to test the feasibility of a novel, evidence-informed strategy that uses a persuasive, informational SMS text message reminder system to expand the impact of secure storage and disposal programs. Our central hypothesis is that the implementation of an SMS text message intervention will increase the secure storage of opioid medication during treatment and disposal following treatment. The intervention will be tested with a 2-arm, single-blinded, RCT design. Participants in the intervention group will receive a series of 7 SMS text messages about securely storing prescription opioids during treatment and disposing of unused prescription opioids. The SMS text messages have been developed and refined by end users of the intervention [31].

The status quo is to encourage secure storage and disposal of unused prescription opioids with the implementation of disposal programs [28,29], community-wide awareness campaigns [32], educational pamphlets [20,21], and drug deactivation products [29,33] delivered by medical providers. Current research indicates the need for improved interventions that effectively facilitate secure storage and disposal of unused prescription opioids. Our study adopts an evidence-based intervention strategy, SMS text messages on mobile phones, for a novel purpose—facilitation of secure storage and disposal of prescription opioids. We use a theoretically driven and user-derived messaging delivered in an SMS text message intervention during a critical window of need following receipt of a prescription opioid medication. The findings from the proposed study have the potential to be scalable across multiple systems of care and expand new horizons for medical systems to use existing digital technologies to improve patient care.

### Limitations

Several limitations may impact this study. Individuals will be eligible to participate within 14 days of receiving their opioid medications. Thus, individuals with shorter prescriptions may receive SMS text messages about secure storage after they have completed treatment and receive SMS text messages about disposal after they have already disposed of their unused medication. We will only recruit individuals who have an opioid prescription for 30 days or less, so findings may not be generalizable to patients who have more than a 30-day prescription. We will not be able to control exposure to external messaging about storing or disposing of opioid medications, but we will be assessing self-reported exposure to messaging. While we will be able to track the delivery of all SMS text messages, we cannot assess if they are received or read by the study participants. A systematic, 2-phase approach was used to refine the text messages for the SMS text message intervention using both focus groups and a Qualtrics (Silver Lake) panel [31]. It is possible, but unlikely, that the SMS text messages will not resonate with or be well received by the participants in the RCT. The RCT is being conducted during an election cycle. Participants may be getting more SMS text messages than usual during this period, which may detract from their attention to the study text messages.

### Conclusion

Upon successful completion of the study, we will have developed and pretested a systems-level, scalable intervention using mobile technology for the secure storage and disposal of unused prescription opioids, which could be implemented in pharmacies and other medical systems. This contribution is expected to be significant in that facilitating secure storage and disposal of unused prescription opioids should reduce the accumulation of these medications that would otherwise be accessible for nonmedical use. Due to the decreased availability of unused prescription opioids, we would expect to see a decline in the prevalence of nonmedical prescription opioid use and associated consequences. Without the identification of strategies that effectively and universally facilitate secure storage and disposal of unused opioid medications, opioid medications with misuse potential will remain in communities, increasing the likelihood for nonmedical prescription opioid-related morbidity and mortality.

## Abbreviations

FDA: US Food and Drug Administration
HBM: Health Belief Model
IRB: institutional review board
ITT: intent-to-treat
RCT: randomized controlled trial
REDCap: Research Electronic Data Capture

## Data Availability

Deidentified data collected during this study are available from the corresponding author upon reasonable request.
